# Heuristic Algorithms for Assigning Hispanic Ethnicity

**DOI:** 10.1371/journal.pone.0055689

**Published:** 2013-02-06

**Authors:** Francis P. Boscoe, Maria J. Schymura, Xiuling Zhang, Rachel A. Kramer

**Affiliations:** 1 New York State Cancer Registry, New York State Department of Health, Albany, New York, United States of America; 2 New York State Cancer Services Program, New York State Department of Health, Albany, New York, United States of America; Harvard School of Public Health, United States of America

## Abstract

We compared several techniques for assigning Hispanic ethnicity to records in data systems where this information may be missing, variously making use of country of origin, surname, race, and county of residence. We considered an algorithm in use by the North American Association of Central Cancer Registries (NAACCR), a variation of this developed by the authors, a “fast and frugal” algorithm developed with the aid of recursive partitioning methods, and conventional logistic regression. With the exception of logistic regression, each approach was rule-based: if specific criteria were met, an ethnicity assignment was made; otherwise, the next criterion was considered, until all records were assigned. We evaluated the algorithms on a sample of over 500,000 female clients from the New York State Cancer Services Program for whom self-reported Hispanic ethnicity was known. We found that all approaches yielded similarly high accuracy, sensitivity, and positive predictive value in all parts of the state, from areas with very low to very high Hispanic populations. An advantage of the fast and frugal method is that it consists of a small number of easily remembered steps.

## Introduction

Race and Hispanic ethnicity are routinely used in public health and public policy analyses in the United States, particularly to identify disparities. They are generally considered useful categories, despite problems with standard definitions and terminology and more fundamental concerns over what identified disparities truly signify [1]–[2]. Most researchers do agree that race and ethnicity data stand to be improved [3]–[6]. For example, despite significant effort over four years, a large health maintenance organization (HMO) was able to obtain race/ethnicity information on only one-third of its enrollees [7]. Similarly, in some state cancer registries, more than half of the records are missing values for ethnicity, although nationally the figure is closer to five percent [8]. Another study found that as of 2008, just twenty states collected hospital discharge data in accordance with current federal race/ethnicity definitions, nineteen followed earlier definitions, and eight did not collect race and/or ethnicity at all [9].

Investigators have tackled the problem of missing race and ethnicity data by trying to make improvements in initial data collection or by linking to external databases [7], [10]. With either of these approaches, the aim is to obtain the self-reported value, which is generally taken to be the best measure of race and ethnicity [11]. Another alternative is to assign a likely value based on the values of other highly predictive variables such as birth place, surname, or residential location [7], [12]–[15]. This approach necessarily introduces some misclassification compared with self-reported values, but can be considerably less costly, as it just involves applying a model or algorithm to already-collected data.

Surnames have long been used to aid in ethnic identification in the United States. The United States Census Bureau has been publishing Spanish surname lists since the 1950 census, with steady improvements in their quality and scope [16]. Morgan and others found the 1990 list to be more predictive of Hispanic ethnicity than codes collected directly by the Medicare program [17]–[18]. The lists have led to key insights; for example, Smith and Bradshaw found that using surnames to calculate mortality rates by ethnicity in Texas partially explained the “Hispanic paradox” by which Hispanics have lower mortality than non-Hispanic whites [19]. Because Hispanic ethnicity is underreported on death certificates, Hispanic mortality rates appear artificially low; using surname-derived ethnicity corrects for this.

In this paper, we evaluated four approaches for assigning Hispanic ethnicity by comparing the results to a large set of self-reported values (Table 1). First, we considered the NAACCR Hispanic Identification Algorithm (NHIA) currently in use by central cancer registries in the United States [13]. This algorithm was developed by a team of over a dozen researchers between 2001 and 2003 and has been subjected to ongoing evaluation and occasional minor adjustment since that time. Next, we considered a data-driven solution developed by the authors (hereafter referred to as the “authors’ method”) based on our experience and familiarity with the data set. We then considered a “fast and frugal” algorithm suggested by the recursive partitioning method [20]–[21]. Recursive partitioning is a technique that creates a decision tree that attempts to maximize the classification of the population based on dichotomous dependent variables. Finally, we considered a traditional logistic regression approach. The four approaches variously made use of birthplace, surname and/or maiden name, race, and county of residence. Each approach classified all records as either Hispanic or non-Hispanic and did not leave any unclassified. All but the regression method are examples of “take the best” heuristics, where a series of criteria are applied, and the process halted as soon as a discrimination is able to be made [22].

**Table 1:** Hispanic identification algorithms evaluated. Number of persons classified by each step given in parentheses.

Algorithm Name	Description
NAACCR Hispanic Identification Algorithm (NHIA)^a^	1. Persons born in non-Spanish-speaking countries in South America and Europe and several other specified countries are coded as non-Hispanic (28,191).
	2. Persons born in Spanish-speaking countries are coded as Hispanic (148,698).
	3. Persons with American Indian, Asian, or Pacific Islander race are coded as non-Hispanic (51,063).
	4. Female maiden names that are Hispanic among at least 75% of the population are coded as Hispanic (7,044).
	5. Female maiden names that are Hispanic among less than 5% of the population are coded as non-Hispanic (68,459).
	6. Female surnames that are Hispanic among at least 75% of the population are coded as Hispanic (14,977).
	7. Remaining cases are coded as non-Hispanic (228,139).
Authors’ algorithm	1. Persons with Asian race are coded as non-Hispanic (51,401).
	2. Persons born in Spanish-speaking countries are coded as Hispanic (148,554).
	3. Persons born in all remaining countries except U.S., Brazil, Portugal (including Cape Verde), and Belize are coded as non-Hispanic (53,219).
	4. Surnames that are Hispanic among at least 75% of the population are coded as Hispanic (20,847).
	5. Surnames that are Hispanic among less than 25% of the population are coded as non-Hispanic (268,497).
	6. Persons from high-Hispanic counties (≥10% Hispanic in the 2000 U.S. census) are coded as Hispanic (1,330).
	7. Persons from low-Hispanic counties (<5% Hispanic in the 2000 U.S. census) are coded as non-Hispanic (671).
	8. Majority-Hispanic surnames are coded as Hispanic (1,194).
	9. Remaining cases are coded as non-Hispanic (858).
Fast and frugal (3-step version)	1. Persons born in Spanish-speaking countries are coded as Hispanic (148,719).
	2. Majority-Hispanic surnames are coded as Hispanic (25,222).
	3. Remaining cases are coded as non-Hispanic (372,630)
Fast and frugal (4-step version)	1. Persons with Asian or Pacific Islander race are coded as non-Hispanic (51,401).
	2. Persons born in Spanish-speaking countries are coded as Hispanic (148,554).
	3. Majority-Hispanic surnames are coded as Hispanic (24,272).
	4. Remaining cases are coded as non-Hispanic (322,344).
Logistic regression	Hispanic ethnicity is a function of country of birth, surname percent Hispanic (using the same categories as in Table 3), county percent Hispanic (grouped into 5% intervals up to 25–30% and over 30%), and race.

For all but the NHIA algorithm, maiden names are used in place of surname when available.

a This is a “female only” version of the published algorithm; a data set including males would require one additional step.

We applied our methods to a sample of female clients from the New York State Cancer Services Program (CSP). The CSP, which is funded through the Centers for Disease Control and Prevention’s (CDC) National Breast and Cervical Cancer Early Detection Program and New York state funds, provides free cancer screening and diagnostic services for uninsured and underinsured age-eligible adults with household incomes less than or equal to 250 percent of the federal poverty level. Demographic and other background information are collected for all clients receiving screening or diagnostic services through the CSP as part of a standard intake process. Clients are asked to self-identify their race and ethnicity as well as their place of birth.

The sample from the CSP included over 500,000 women served between 1994 through 2010, over 180,000 of whom self-identified as Hispanic (Table 2). This ratio of nearly one-third Hispanic was considerably higher than the Hispanic proportion in New York State as a whole for this period, about 13 percent. Participants in this program were also more likely to be Asian, foreign born, and between the ages of 40 and 59 than the overall population. The data set was geographically balanced, however, with ample representation from all parts of the state, including low-population, low-Hispanic counties.

**Table 2:** Comparison of New York State and CSP populations, age 18 and above.

		New York State, 2000 Census (%)	CSP, 1994–2010 (%)^a^
Race/ethnicity	Hispanic	13.3	32.5
	White	61.7	39.9
	Black	14.7	15.9
	Asian	5.4	9.4
	Other	4.8	2.3
Birthplace	Born in U.S.	72.2	49.5
	Born in Spanish-speaking country	9.7	27.5
	Other foreign-born	18.1	23.0
Age	18–39	40.7	19.8
	40–49	19.4	33.4
	50–59	15.0	25.9
	60–69	10.2	13.3
	70–79	8.8	5.7
	80+	5.9	1.9
Geography	New York City	43.3	43.4
	New York State Excluding New York City	56.7	56.6

a Excludes records with missing information, which ranged from 0 percent (age) to 3 percent (birthplace).

## Data and Methods

Using the four approaches presented in Table 1, we derived Hispanic ethnicity for 546,571 unique women from the CSP client database from the years 1994 to 2010 with known self-reported Hispanic ethnicity. An additional 16,961 persons with unknown Hispanic ethnicity were excluded from the analysis. Birthplace consisted of the state, territory, or country of birth, coded using standard cancer registration codes that group some less common birthplaces together, such as some Pacific Island countries [23]. Spanish-speaking birthplaces were defined as Argentina, Bolivia, Chile, Colombia, Costa Rica, Cuba, Dominican Republic, Ecuador, El Salvador, Guatemala, Honduras, Mexico, Nicaragua, Panama, Paraguay, Peru, Puerto Rico, Spain, Uruguay, and Venezuela. Non-specific birthplace codes for Central America, South America, and Latin America (n = 271) were also counted in the Spanish-speaking group. Equatorial Guinea is the only other country with Spanish as an official language, but no separate code existed for this country, as it was grouped with those from other West African nations.

Birthplace was not specified for 2.9% of the sample. For these records, algorithmic rules regarding birthplace (i.e., whether born in a Spanish-speaking country) were always taken to be false. Analogous reasoning was used for persons missing race (4.7%) and/or county (0.5%).

Hispanic surnames were determined using a list of 151,671 surnames occurring at least 100 times in the United States in the 2000 census, tabulated by race and ethnicity [24]. This list, released in 2008, is based on 86 percent of the entire U.S. population. As names on this file are limited to 14 characters, the match to the CSP data was based on the first fourteen characters; just 0.2 percent of the names on the CSP file contained 15 or more characters. Following a practice developed after the 1990 census, names were grouped into five categories based on the likelihood that a given surname was reported as Hispanic (Table 3) [18]. Names not on the list (i.e., those occurring fewer than 100 times) were counted as rarely Hispanic. Where both surname and maiden name were available, the maiden name was used, except in the NHIA method where both were considered in certain instances (see Table 1).

**Table 3:** Hispanic Surname Classification Scheme.

Designation	% of Persons	Number of Names,2000 Census (%)	Number of Hispanicswith this designation,2000 Census (%)	Number of Hispanicswith this designation,CSP data (%)
Heavily Hispanic	≥75%	6,020 (4.0)	25,353,317 (71.8)	129,839 (73.9)
Generally Hispanic	≥50%–<75%	1,774 (1.1)	1,185,327 (3.4)	9,627 (5.5)
Moderately Hispanic	≥25%–<50%	1,616 (1.1)	429,309 (1.2)	3,748 (2.1)
Occasionally Hispanic	≥5%–<25%	11,179 (7.4)	547,786 (1.6)	4,236 (2.4)
Rarely Hispanic	<5%	131,082 (86.2)	7,790,079 (22.1)	28,166 (16.0)
Total		151,671 (100.0)	35,305,818 (100.0)	175,616 (100.0)

Derived Hispanic ethnicity was compared with self-reported Hispanic ethnicity for each of the four methods, and accuracy, sensitivity, specificity, positive predictive value (PPV), negative predictive value (NPV), and relative bias were calculated. Relative bias is defined as the percent underprediction or overprediction of Hispanics relative to the true number. Two different versions of the fast and frugal method were tabulated, one using three steps and one using four steps. Results were tabulated for the entire population as well as for three different levels of Hispanic prevalence: counties less than 5 percent Hispanic, 5 to 10 percent Hispanic, and over 10 percent Hispanic, as reported in the 2000 census. This step was taken to verify that the results were applicable to all regions of the state.

The logistic regression model computed a probability of each individual being Hispanic based on race (white, black, American Indian, Asian, Pacific Islander, other, unknown), birthplace (United States, Spanish-speaking country, Brazil or Portugal, other North American country, other South American country, other European country, other), Hispanic surname prevalence (as listed in Table 3), and county of residence (in 5 percent increments, from 0–5 to 30+ percent). Persons with probabilities of 0.3 or greater were counted as Hispanic; this probability matched the population prevalence and yielded the most accurate results. To minimize the impact of overfitting and increase the model’s generalizability to data from other states, we partitioned the state into two areas with similar population size, Hispanic prevalence, and a mixture of urban and rural areas. The first area consisted of 46 counties and included the New York City boroughs of Manhattan and the Bronx, the northern suburbs of New York City, and most of upstate New York, including the urban centers of Syracuse and Buffalo. The second area consisted of 16 counties and included the New York City boroughs of Brooklyn, Queens, and Staten Island, Long Island, and north-central New York including Rochester. We then performed two-fold cross validation, modeling each of the areas and testing with the other, and summing the two results.

## Results

Table 4 lists self-reported ethnicity versus algorithm-derived ethnicity for each of the methods, first for the entire data set and then stratified by county Hispanic prevalence. Each of the methods did similarly well at classifying Hispanics. For the entire data set, accuracy ranged from 96.3 to 96.6 percent and sensitivity from 92.9 to 93.9 percent, while specificity, PPV, and NPV showed similarly high values and low variability. All methods slightly underestimated the actual number of Hispanics, ranging from 1.0 percent to 2.8 percent, as indicated by the relative bias measure. With the exception of regression, each method performed the best overall by at least one of the six quality measures. Greater variation was seen after stratifying results into counties with high, medium, and low Hispanic prevalence. In medium and low Hispanic counties, sensitivity and PPV were reduced, while specificity and NPV rose. In other words, proportionally more true Hispanics were missed and true non-Hispanics were counted as such in these counties. This in turn resulted in higher overall accuracy and more pronounced relative bias. These effects were particularly noticeable for the regression method. The relative bias finding contradicts a result from Minnesota which showed that NHIA resulted in an overestimation of Hispanics in low-Hispanic counties, though this effect was only seen in counties below 2 percent Hispanic [13].

**Table 4:** Hispanic Classification Results by Method and County Hispanic Prevalence.

		Self-Reported Value/Algorithm-Derived Value				Quality Measure^b^					
County HispanicPrev.^a^	Method	Hispanic/Hispanic (A)	Hispanic/Non-Hispanic (B)	Non-Hispanic/Non-Hispanic (C)	Non-Hispanic/Hispanic (D)	Acc	SN	SP	PPV	NPV	RB
All	NHIA	163,175	12,441	363,411	7,544	96.3	92.9	98.0	95.6	96.7	−2.8
	Authors’	164,596	11,020	363,626	7,329	96.6	93.7	98.0	95.7	97.1	−2.1
	FF (4)	164,800	10,816	362,929	8,026	96.6	93.8	97.8	95.4	97.1	−1.6
	FF (3)	164,900	10,716	361,914	9,041	96.4	93.9	97.6	94.8	97.1	−1.0
	Regression^c^	163,694	11,922	363,193	7,762	96.4	93.2	97.9	95.5	96.8	−2.4
High	NHIA	140,402	8,987	170,050	5,176	95.6	94.0	97.0	96.4	95.0	−2.6
	Authors’	141,323	8,066	170,188	5,038	96.0	94.6	97.1	95.6	95.5	−2.0
	FF (4)	141,803	7,586	169,405	5,821	95.9	94.9	96.7	96.1	95.7	−1.2
	FF (3)	141,839	7,550	168,593	6,633	95.6	94.9	96.2	95.5	95.7	−0.6
	Regression	140,748	8,641	169,277	5,949	95.5	94.2	96.6	95.9	95.1	−1.8
Medium	NHIA	16,600	1,666	46,664	982	96.0	90.9	97.9	94.4	96.6	−3.7
	Authors’	16,705	1,561	46,774	872	96.3	91.5	98.2	95.0	96.8	−3.8
	FF (4)	16,768	1,498	46,692	954	96.3	91.8	98.0	94.6	96.9	−3.0
	FF (3)	16,772	1,494	46,600	1,046	96.2	91.8	97.8	94.1	96.9	−2.5
	Regression	17,165	1,101	46,443	1,203	96.5	94.0	97.5	93.5	97.7	0.6
Low	NHIA	5,199	1,621	145,137	1,347	98.1	76.2	99.1	79.4	98.9	−4.0
	Authors’	5,114	1,706	145,510	974	98.2	75.0	99.3	84.0	98.8	−10.7
	FF (4)	5,268	1,552	145,220	1,264	98.2	77.2	99.1	80.6	98.9	−4.2
	FF (3)	5,275	1,545	145,178	1,306	98.1	77.3	99.1	80.2	98.9	−3.5
	Regression	4,714	2,106	146,073	411	98.4	69.1	99.7	92.0	98.6	−24.9

a County Hispanic prevalence: High: 9 counties with ≥10% Hispanic population according to the 2000 U.S. Census; Medium: 7 counties with 5–10%; Low: 46 counties with <5% Hispanic. In the CSP sample, 149,389 of 324,615 from high-Hispanic counties self-identified as Hispanic (46%). Corresponding values for medium-Hispanic counties were 18,266 of 65,912 (28%), and for low-Hispanic counties were 6,820 of 153,304 (4%). County was not known for 2,740 persons.

b Quality measures: Acc: Accuracy (A+C)/(A+B+C+D); SN: Sensitivity (A)/(A+B); SP: Specificity (C)/(C+D); PPV: Positive Predictive Value (A)/(A+D); NPV: Negative Predictive Value (C)/(B+C); RB: Relative Bias [(A+D)/(A+B)]−1.

c Sum of the two models, each containing approximately half of the observations.

We do not report statistical tests on the differences between the methods because their interpretation is unclear: given the large sample sizes, the small differences seen would tend to rate as “significant” based on sampling theory, but non-sampling error (i.e., data entry and transcription error) is likely a more important source of variation in this data set. Also, given the differences in the underlying ethnic compositions of states, these results for New York should be considered merely good approximations and not precise predictors of what could be expected in other states.

## Discussion

Given the similar results from the different algorithms, no single option stands out as clearly superior. Simplicity is therefore an important consideration for selecting among them. There are a number of reasons to prefer a simpler algorithm. First, it facilitates code maintenance, from the need for periodic updates (as when a new surname list becomes available) to the need for local modification (as in Hawaii, where the legacy of Spanish colonization of parts of the Pacific requires needs to be taken into account). Simpler code also makes for simpler translation into other programming languages. Second, a simpler algorithm is more easily comprehended and communicated. The first author of this paper serves as the technical contact for the NHIA algorithm, and based on the number of detailed questions he has received over the years, he can attest to a broad preference for transparency and clarity among users. Lastly, simpler algorithms are often more predictive than complex algorithms when applied to new locations or time periods. This is because complex algorithms are more susceptible to the problem of overfitting, either because they incorporate information unique to the test data set or because they insufficiently distinguish pattern from noise [15], [25].

Given these considerations, the fast and frugal approach is particularly attractive. The rules (if not the specific surnames) can be committed to memory, summarized on an index card, or readily adapted into any computer language. There are, at most, just three questions to ask: Was the person born in a Spanish-speaking country? Does he or she have a Spanish surname? Is he or she Asian or Pacific Islander? The authors’ and NHIA methods, in contrast, provide evidence of the diminishing returns of added complexity. While each step in the authors’ method resulted in a better fit to the self-reported values, by step 6 the magnitude of these improvements had become negligible. For NHIA, the intricate rules involving maiden name seem reasonable but offered little gain, while the counting of American Indians and Brazilians as non-Hispanic actually reduced the overall accuracy, as they reflect dated notions that these categories are mutually exclusive (data not shown).

The county-stratified results support the use of a single rule applicable to all locations, regardless of the underlying Hispanic prevalence. To the extent that there were differences, low-Hispanic counties traded lower sensitivity and PPV for higher specificity and NPV, but had higher overall accuracy. This is because the comparatively few Hispanics in these counties were somewhat more difficult to detect. For example, 98 percent of over 2,000 women in high-prevalence Hispanic counties with the birth name Gonzalez self-reported as Hispanic, while in low-prevalence counties the figure was 88 percent of 150 women. The name was a good predictor in both instances, just not an equally good predictor. Hispanic self-identity is not exclusively a function of ancestry, but is also a dynamic construction of interactions with family, neighbors, and community - items that can never be fully captured by an algorithm.

A more striking example was seen among persons born in Brazil, Portugal, and Cape Verde. According to the usual federal definition, these persons are non-Hispanic, because they are neither of “Spanish speaking background” nor “have origins in Spanish-speaking countries” [26]. In our sample, however, 32 percent of Portuguese and 46 percent of Brazilians identified themselves as Hispanic. Coincidentally, nearly half of the Brazilian and Portuguese surnames appeared on the Hispanic surname list, so applying the list to these groups yielded close to the correct number of Hispanics overall. While not accurate at the individual level, this yielded a better overall result than if they had been counted as either entirely Hispanic or entirely non-Hispanic. (In a state such as Massachusetts where Portuguese speakers greatly outnumber Spanish speakers, this approach would require more scrutiny). Comparable results to ours have been found in the U.S. census, even though the past three censuses have attempted to discourage Portuguese speakers from identifying as Hispanic by including the term “Spanish” wherever “Hispanic” appears on a form [27].

There was also variation in Hispanic self-identification within Spanish-speaking countries. While most were near 100 percent, there were two outliers: Spain (81 percent) and Panama (64 percent). When developing the authors’ method, we considered making a special rule governing these countries, but ultimately did not given that they represented just 0.1 percent of the sample. This is just one of many possible narrowly focused additional rules that we could have included that would have resulted in marginal gain and increased likelihood of overfitting.

A potential limitation of all of the methods is that while the CSP data set had the advantages of being large and geographically diverse, it is not representative of the population as a whole, either of New York State or the United States. If Hispanic self-identification varies significantly between lower-income women and higher-income women, or between women and men, or between New Yorkers and non-New Yorkers, then similar results might not be obtained when these methods are applied to a wider population. However, the fact that the NHIA algorithm has been applied to cancer patients nationwide for nearly a decade ameliorates this concern. We further note that the CSP is a public health program and not a study where data are collected through a rigorous research protocol. A large number of clinical and program staff have been responsible for collecting the ethnicity and country of origin data for the CSP clients over the years. While we believe the data to be of good quality, their accuracy has not been assessed or verified.

Finally, we note that the assignment of ethnicity (or race, or any other demographic or clinical variable in public health surveillance) is typically done on a small fraction of cases for which the value is unknown, not on an entire population, as we did here. For a more typical real-world example, imagine a data set with 20 percent of the records coded as Hispanic and 10 percent coded as unknown ethnicity, and assume that the various algorithms designate between 24 and 25 percent of the unknown as Hispanic. The resulting Hispanic prevalence in the data set would range from 24.4 to 24.5 percent. The impacts on disease rates or other secondary outcomes of interest would be of a similar range. This further argues for an approach that is simple and memorable.
